# A solitary brain metastasis from gastroesophageal junction adenocarcinoma 13 years after curative multimodality treatment: a case report and review of the literature

**DOI:** 10.3389/fonc.2026.1892637

**Published:** 2026-10-16

**Authors:** Sara Di Lorenzo, Pasqualina Giordano, Giuseppe Catapano, Francesca Maria Giugliano, Piera Federico, Federico Frio, Antonio D’Antonio, Bruno Daniele, Angelica Petrillo

**Affiliations:** 1 Medical Oncology, Department of Precision Medicine, University of Campania “Luigi Vanvitelli”, Naples, Italy; 2 Medical Oncology Unit, “Cav. R. Apicella” Hospital, Pollena Trocchia, Italy; 3 Medical Oncology Unit, Ospedale del Mare, Naples, Italy; 4 Neurosurgery Unit, Ospedale del Mare, Naples, Italy; 5 Radiation Oncology Unit, Ospedale del Mare, Naples, Italy; 6 Pathology Unit, Ospedale del Mare, Naples, Italy

**Keywords:** blood-brain barrier, brain metastasis, gastroesophageal adenocarcinoma, multidisciplinary care, tumour dormancy

## Abstract

Brain metastases in gastroesophageal adenocarcinoma are uncommon and can cause substantial neurological morbidity. Here, we report the case of a 58-year-old patient who developed a solitary brain metastasis thirteen years after curative-intent multimodality treatment for a Siewert type II gastroesophageal junction adenocarcinoma (GEJA). The intracranial lesion was initially radiologically suspected to be a primary brain tumour, while systemic restaging showed no extracranial disease. Histopathology, immunohistochemistry, clinical history, and methylome profiling supported metastatic recurrence from the prior GEJA. This case is noteworthy for the exceptionally long disease-free interval, the brain-only presentation after routine oncological surveillance had ended, and the diagnostic importance of tissue confirmation and integrated pathological assessment when a new intracranial lesion appears many years after curative treatment. This case supports maintaining late metastatic recurrence in the differential diagnosis of symptomatic brain lesions without implying a role for routine neuroimaging in asymptomatic long-term survivors.

## 1 Introduction

Gastric cancer (GC) remains one of the leading causes of cancer-related mortality worldwide. According to global cancer statistics (1), it ranks fifth both in incidence, with 968,350 new cases, and in mortality, accounting for 659,853 deaths. Surgery is the only potentially curative treatment for localised disease, although recurrence rates remain high even after radical resection (2). For this reason, perioperative chemotherapy has been introduced to improve outcomes, particularly in locally advanced disease (2). Most recurrences occur within the first two years, whereas late relapses beyond five years are uncommon (3).

Despite advances in treatment, many patients with GC gastric cancer are diagnosed with metastatic disease, which is associated with poor survival (4). Common metastatic sites include the liver, lymph nodes, peritoneum, lungs, and bones (5). Brain metastases from GC are uncommon, historically reported in <1% of patients (4, 6). Brain involvement is also uncommon after curative treatment of oesophageal and gastroesophageal junction adenocarcinoma (GEJA). However, contemporary surgical cohorts have reported brain tumours or solitary brain metastases in approximately 2-3.4% of patients (7–9). Differences in primary site, histology, stage, neuroimaging practices, and case ascertainment limit direct comparison across these populations.

In this context, we describe the case of a patient with a solitary brain metastasis arising thirteen years after curative treatment for a Siewert type II GEJA and provide a focused narrative review of late and isolated intracranial recurrence. The junctional location warrants specific consideration: available genomic data indicate substantial overlap between Siewert type I and II adenocarcinomas and lower oesophageal adenocarcinoma, whereas Siewert type III tumours show more gastric features (10). Accordingly, we interpret evidence from both GC gastric and GEJA literature.

## 2 Case report

### 2.1 Clinical presentation

In September 2021, a 58-year-old Caucasian man presented to the emergency department of Ospedale del Mare (Naples, Italy) after a seizure involving the left upper limb.

A non-contrast brain computed tomography (CT) scan revealed a 30 mm lesion in the right parietal region, surrounded by oedema, suggestive of a primary brain tumour.

Subsequent contrast-enhanced magnetic resonance imaging (MRI) confirmed the presence of a solid lesion (29 × 26 mm) in the right parietal lobe, associated with extensive oedema involving the frontoparietal and temporal regions and mild ventricular compression (**Figure 1**). Thus, the patient was admitted to the neurosurgical unit for further evaluation.

**Figure 1:**
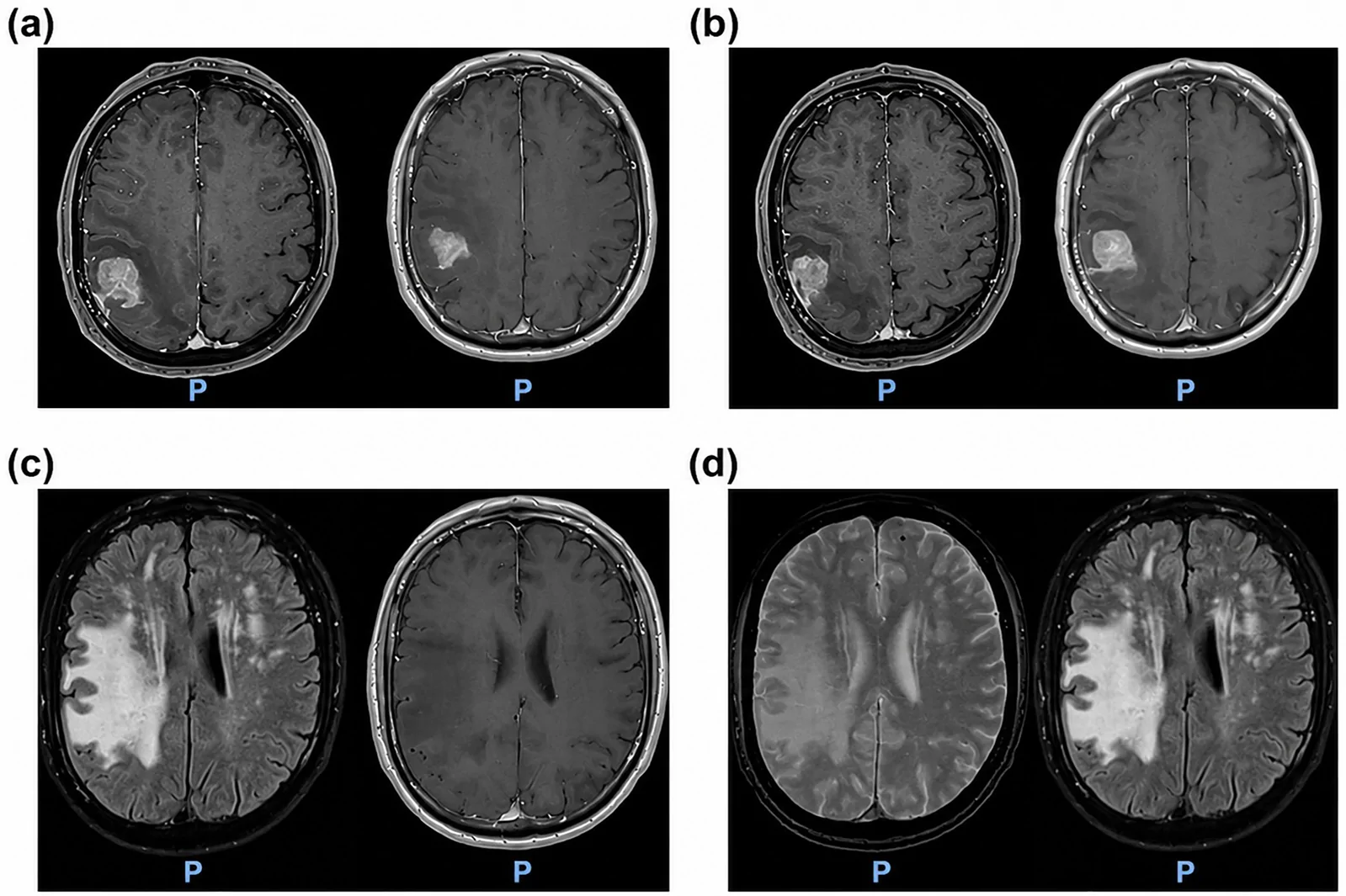
Representative magnetic resonance imaging (MRI) images of the lesion at the diagnosis (September 2021). **(a)** Axial T1-weighted images before and after gadolinium administration. **(b)** Additional contrast-enhanced T1-weighted images showing lesion enhancement. **(c)** FLAIR and post-contrast T1-weighted images demonstrating the lesion and the associated vasogenic oedema. **(d)** Axial FLAIR images illustrating the extent of the surrounding oedema.

### 2.2 Clinical history

His medical history included hypertension, bilateral hernioplasty, and Pseudomonas aeruginosa pneumonia in June 2021 without sequelae. He was a former smoker (approximately 2 packs/day until the age of 30).

The patient reported an oncological history that began in 2008, when he was diagnosed with GEJA (Siewert type II). Preoperative staging with oesophagogastroduodenoscopy (EGDS), contrast-enhanced total-body CT, and 18F-fluorodeoxyglucose positron emission tomography (18F-FDG PET) showed localised disease, and tumour markers (CEA and CA 19-9) were normal.

He underwent total gastrectomy with D1+ lymphadenectomy with curative intent at another institution. Pathological staging was pT3N1 (1/18 lymph nodes), with no distant metastases and a well-differentiated tumour. Human epidermal growth factor receptor 2 (HER-2) testing was not performed because it was not standard for localised disease at that time.

From April to September 2009, he received 12 cycles of adjuvant modified FOLFOX-4 chemotherapy at another institution, which was well tolerated without grade 3–4 adverse events.

He then underwent regular follow-up for ten years with no evidence of recurrence; routine oncological surveillance was discontinued in 2019.

### 2.3 Diagnosis and treatment

During hospitalisation, the patient received dexamethasone and levetiracetam, which resolved symptoms. His Eastern Cooperative Oncology Group (ECOG) performance status (PS) was 0.

After discussion by the brain multidisciplinary team (MDT), he underwent craniotomy for a suspected primary brain tumour in October 2021.

Histological examination unexpectedly revealed metastatic adenocarcinoma morphologically and immunophenotypically compatible with recurrence from the previous primary GEJA (**Figure 2**). Immunohistochemistry (IHC) showed positivity for cytokeratin (CK) 7, CK20, caudal type homeobox 2 (CDX2), and epithelial membrane antigen (EMA); focal positivity for mucin (MUC) 2 and MUC5; and negativity for thyroid transcription factor 1 (TTF1) and glial fibrillary acidic protein (GFAP). HER-2 was scored as 2+, but *in situ* hybridisation (ISH) showed no amplification.

**Figure 2:**
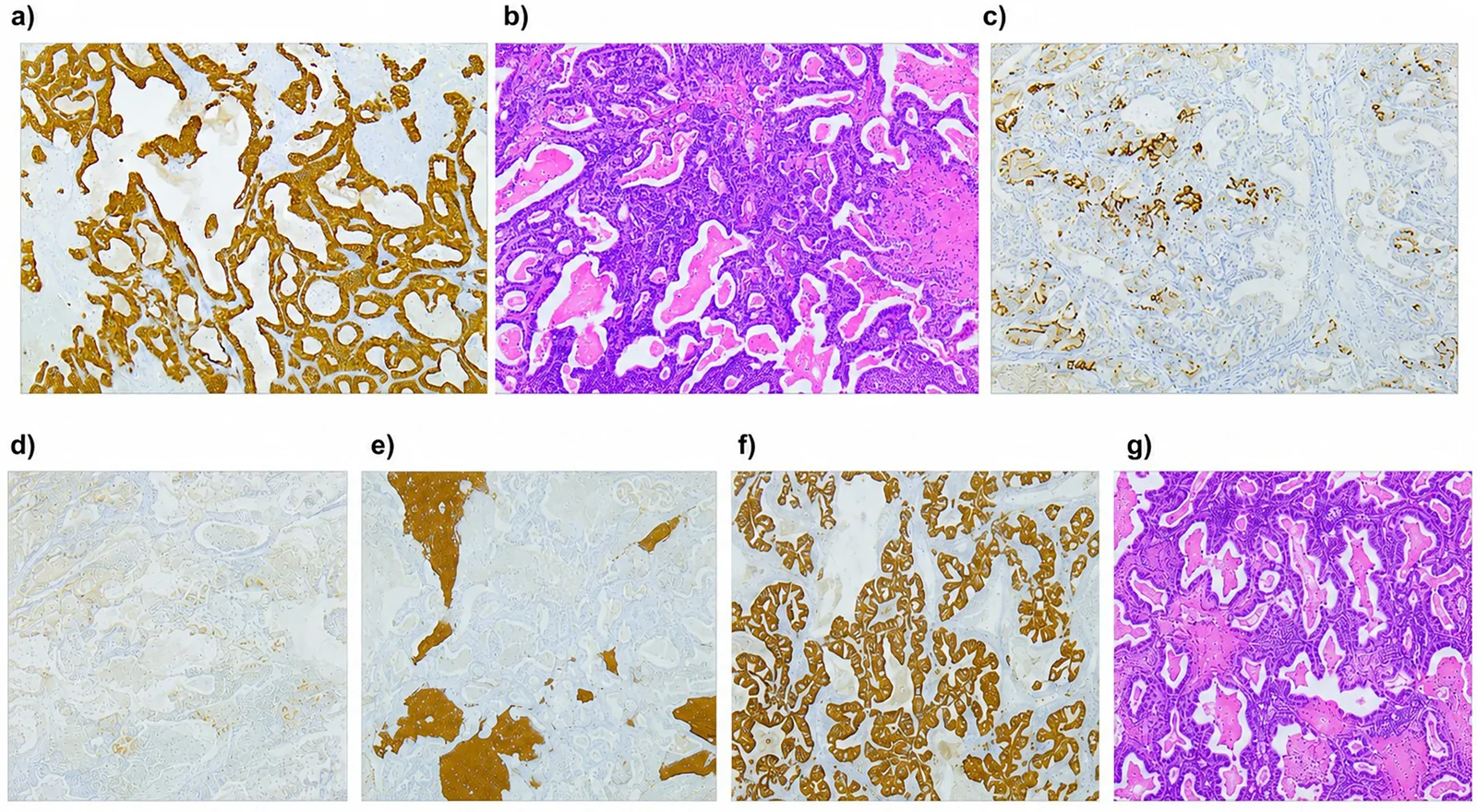
Histological and immunohistochemical features of the tumour. Representative sections stained with **(a)** cytokeratin 20 (CK20), **(b)** haematoxylin and eosin (H&E) first image, **(c)** mucin 2 (MUC2), **(d)** mucin 5AC (MUC5AC), **(e)** glial fibrillary acidic protein (GFAP), **(f)** cytokeratin 7 (CK7), and **(g)** H&E second image.

Molecular analysis by EPIC array, performed according to the institutional protocol for brain tumours, showed no O6-methylguanine-DNA methyltransferase (MGMT) promoter methylation; isocitrate dehydrogenase (IDH) 1 and 2 wild-type status; no 1p/19q co-deletion; and copy-number alterations including partial chromosome 7 amplification, amplification of CDK4, MYC, NF1 and FGFR3, and loss of TP53 and MDM4. Methylome profiling supported the diagnosis of metastatic disease of probable gastric origin.

Attribution of the brain lesion to recurrence from the prior GEJA was based on the integration of morphology, clinical history, IHC, systemic staging, and methylome profiling rather than on a single assay. The CK7/CK20/CDX2/EMA-positive profile with focal MUC expression supported gastrointestinal differentiation, whereas TTF1 and GFAP negativity argued against a pulmonary/thyroid primary and a glial neoplasm, respectively. The absence of another primary tumour on radiological assessment (see below) further supported recurrence from the known GEJA. Methylome profiling provided additional molecular support, although the result was interpreted as supportive rather than definitive because methylation-based classifiers depend on tumour purity, tissue quality, and the available reference classes.

EGDS, CT scan with contrast, brain MRI with contrast, and 18FDG PET total body demonstrated no extracranial disease. Tumour markers remained normal. After the gastric MDT discussion, a strategy of close follow-up was adopted given the long interval since the initial diagnosis and the absence of systemic disease.

In February 2022, MRI findings suggested local recurrence, and the patient underwent fractionated stereotactic radiotherapy to the surgical cavity and adjacent posterior nodule (25 Gy (5Gy/day) and 30 Gy (6Gy/day) in 5 fractions, respectively). Post-treatment MRI showed a response to radiotherapy.

In July 2022, the patient developed mandibular pain (6 according to the numerical pain rating scale (NRS) (11)). Imaging and PET scans showed no evidence of recurrent systemic disease, although CA 19–9 was elevated (83.88 UI/ml). Dental and neurological evaluations were performed. Additionally, an analgesic treatment was started.

In October 2022, the patient underwent a cone-beam CT scan, which highlighted a bone gap involving the lower part of the right mandibular branch. Subsequent MRI demonstrated increased dural thickening, meningeal infiltration, and progression of infiltrative changes around the surgical cavity. Colonoscopy and EGDS were negative. A biopsy of the right mandibular lesion confirmed metastatic GC. Thus, the IHC profile was performed on two specimens from this patient: the metastatic brain lesion resected in 2021 and the new bone biopsy. The results are summarised in **Table 1**. IHC analysis showed HER-2 negativity, PD-L1 combined positive score (CPS) <1, and microsatellite-stable disease in both.

**Table 1:** IHC profile from the patient’s specimens.

Samples	HER-2	PD-L1 CPS*°	Microsatellite status
Specimen from the resection of the metastatic brain lesion (Oct 2021)	2+; ISH not amplified	<1	MSS **
Specimen from the bone biopsy (Dec 2022)	1+	<1	MSS

HER-2, human epidermal growth factor receptor 2; PD-L1, programmed death ligand 1; CPS, combined positive score; MSS, microsatellite stable status; ISH, in situhybridization.

*CPS was defined as follows: the number of positive cells (both tumour and immunity cells) divided by the total number of viable tumour cells, multiplied by 100; ° Ab Roche/Ventana clone SP263 as per local internal procedure; **by immunohistochemistry; this status was also verified by RT-PCR with Idylla-Biocartis automated method.

At reassessment, the patient had ECOG PS 1 and weight loss related to pain-induced feeding difficulties. Nutritional support was initiated for weight loss <10% and a Malnutrition Universal Screening Tool (MUST) score of 1. Multidisciplinary evaluation recommended first-line chemotherapy for stage IV GEJA after updated imaging.

By January 2023, systemic progression involving lymph nodes, liver, and lungs was documented. Based on international guidelines for GC at the time (12), tumour profile, and clinical status, the patient received modified FOLFOX-4 chemotherapy plus denosumab from February to March 2023. Treatment initially produced symptomatic improvement. However, after four cycles, the patient developed pulmonary embolism and rapidly deteriorated clinically. He was transitioned to palliative care at home and died in May 2023.

**Figure 3** summarises the clinical case timeline.

**Figure 3:**
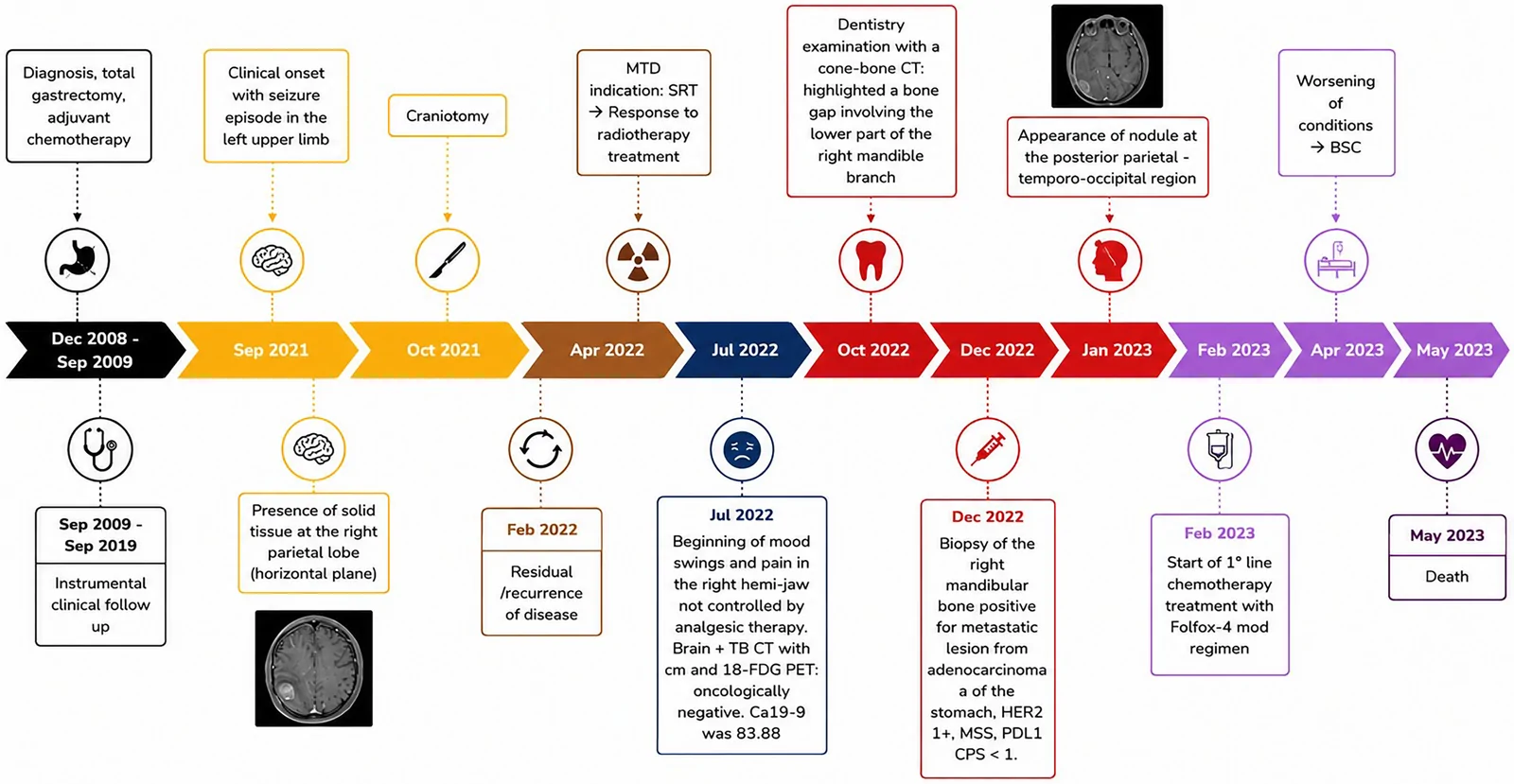
Clinical case timeline. MDT, multidisciplinary team; SRT, stereotactic radiotherapy; TB, total body; CT, computed tomography; 18FDG PET, 18F-fluorodeoxyglucose positron emission tomography; CA 19-9, carbohydrate antigen 19-9; HER-2, human epidermal growth factor receptor 2; PD-L1, programmed death-ligand 1; CPS, combined positive score; MSS, microsatellite stable status; BSC, best supportive care; mod, modified; FOLFOX-4 mod: oxaliplatin 85 mg/m2 on day 1, leucovorin 200 mg/m2 on day 1, fluorouracil 400 mg/m2 as an intravenous bolus on day 1 followed by continuous infusion of fluorouracil 2400 mg/m2 over 46 h.

## 3 Discussion

Brain metastases are uncommon across gastroesophageal adenocarcinomas, but reported incidence varies by primary site and study population. Historical GC series generally reported rates below 1% (6, 13, 14), whereas contemporary cohorts after oesophagectomy have described brain tumours or solitary brain metastases in approximately 2-3.4% of patients (7–9). These figures should not be directly equated because the studies differ in tumour location, histology, stage, treatment, surveillance, and whether pathological confirmation was obtained. The distinction is particularly relevant for a Siewert type II GEJA, which sits at the interface between conventional gastric and oesophageal classifications.

To place our report in context, we performed a focused, non-systematic literature search in PubMed/MEDLINE, Google Scholar, and the reference lists of relevant articles up to 30 June 2026, using combinations of the terms ‘gastric cancer’, ‘gastroesophageal junction’, ‘oesophageal cancer’, ‘brain metastasis’, ‘solitary’, ‘isolated’, ‘late recurrence’, ‘gastrectomy’, ‘esophagectomy’, ‘10 years’, and ‘120 months’. This literature confirms that solitary or isolated brain relapse is not unprecedented: recent esophagectomy cohorts have characterised isolated or solitary brain metastases (7–9), while the gastric literature includes two patients with disease-free intervals exceeding 120 months, a case of isolated brain metastasis 22 years after surgery, and a recent series of 12 patients with solitary intracranial relapse (15, 16). Thus, the contribution of the present case should not be framed as the first observation of this phenomenon or as novelty based on rarity alone.

The added value of this case lies in the convergence of several features with direct diagnostic relevance: a 13-year disease-free interval after curative-intent multimodality therapy, recurrence after routine surveillance had already been discontinued, an initial brain-only presentation that radiologically mimicked a primary brain tumour, and the need to integrate histology, IHC, systemic staging, and methylome profiling to attribute the lesion to the previously treated GEJA. The subsequent course, with later mandibular and systemic metastases, also illustrates how an apparently isolated intracranial event may precede overt extracranial dissemination. The practical message is to retain late metastatic recurrence in the differential diagnosis of a new symptomatic intracranial lesion despite a very long disease-free interval, while recognising that the available evidence does not support routine brain imaging of asymptomatic long-term survivors.

The biological context of the primary tumour is also relevant. A recent genomic analysis of 350 surgically treated GEJA showed that Siewert types I and II were predominantly characterised by chromosomal-instability features and had greater genomic overlap with lower oesophageal tumours, whereas Siewert type III tumours were more heterogeneous and shared more features with GC (10). This supports considering both oesophageal and gastric literature when interpreting a Siewert type II case, but it does not establish a specific biological mechanism for late brain tropism in this individual patient.

The prolonged disease-free interval reported here may be partially explained by tumour dormancy (17, 18). Dormant tumour cells can remain viable in a quiescent state, evade immune surveillance, and resist cytotoxic chemotherapy because they are not actively proliferating. Microscopic residual disease may also remain below the detection threshold of currently available imaging techniques (17, 18) (**Figure 4**).

**Figure 4:**
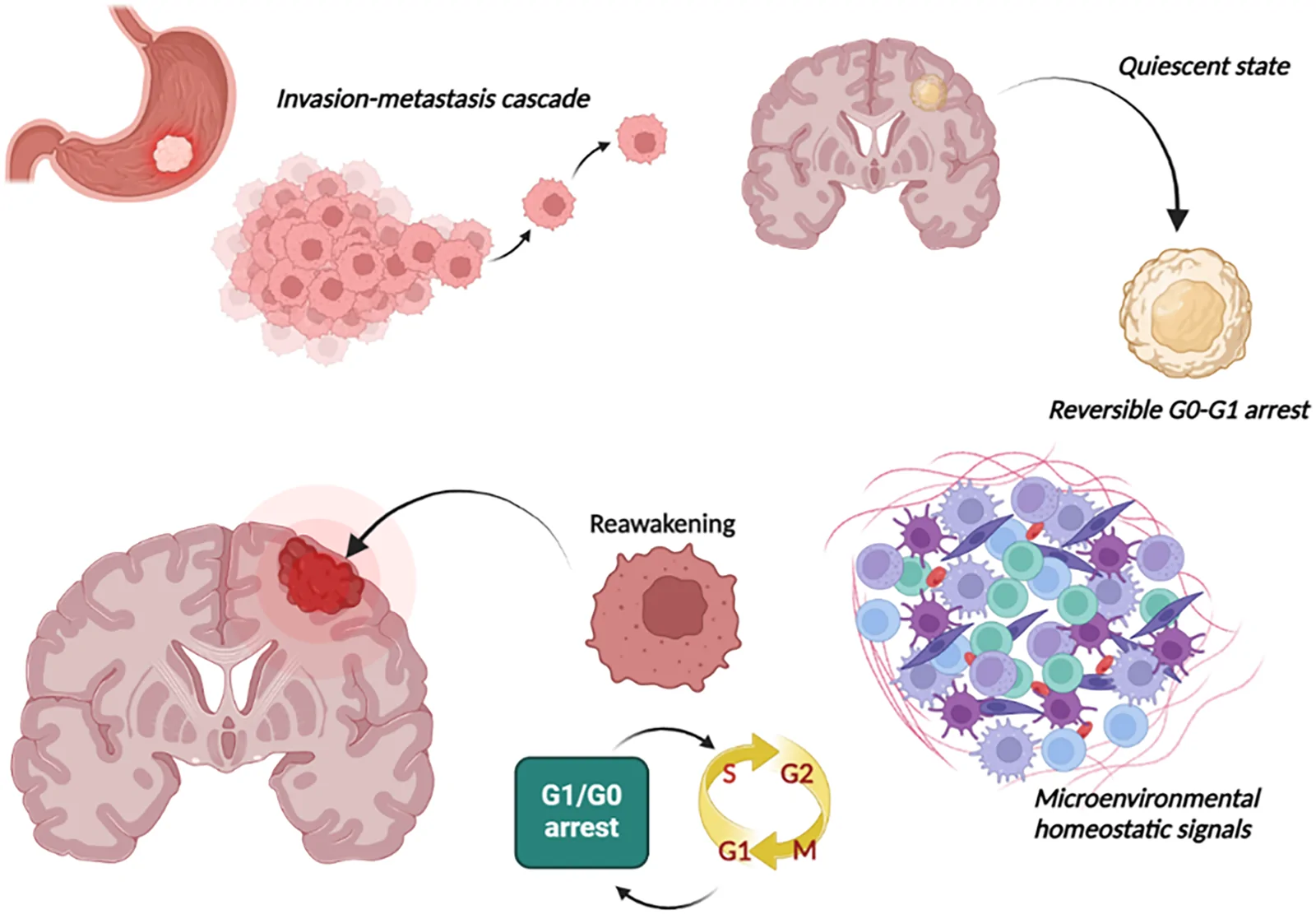
Schematic representation of the tumour dormancy theory. The figure illustrates dissemination from the primary GC tumour, a quiescent phase with reversible G0-G1 cell-cycle arrest, and potential later reactivation in response to microenvironmental signals.

Reactivation of dormant cells may occur through incompletely understood mechanisms, including genetic and epigenetic alterations, angiogenic switching, immune escape, and interactions with the tumour microenvironment (19). This interpretation remains speculative in a single case and should not be considered proof of dormancy; rather, dormancy provides one biologically plausible explanation for the unusually long interval between curative-intent treatment and metastatic relapse.

Outcomes of patients with GC and brain metastases are generally poor, although prognosis varies substantially across small and heterogeneous cohorts. Older series reported median overall survival of only a few months (13, 20), whereas selected patients with solitary brain lesions, absence of extracranial disease, good PS, and access to surgery or stereotactic radiotherapy may experience longer survival (17, 21–23). These data should be interpreted cautiously because most studies are retrospective, include small numbers of patients, and combine synchronous and metachronous metastases, different primary tumour sites, and different local and systemic therapies.

No definitive predictive factors for brain metastases from GC have been established. Retrospective studies suggest associations with male sex, younger age, adenocarcinoma histology, advanced nodal stage, high tumour grade, proximal location, HER-2 positivity or amplification, and concomitant lung or bone metastases (4, 15, 16, 20–22, 24). However, these associations are hypothesis-generating and are limited by selection bias, inconsistent screening practices, and the rarity of the event.

The pathological work-up in the present case was strengthened by concordant morphology, IHC, and methylome profiling. Molecular studies comparing primary gastroesophageal tumours and brain metastases have reported frequent HER-2 amplification in metastatic lesions, as well as alterations involving TP53, EGFR, CDKN2A, SMAD4, ARID1A and other pathways (24, 25). In our patient, the HER-2 2+ result in the brain metastasis was ISH non-amplified and was therefore considered negative; the subsequent bone metastasis was HER-2 1 +. This variability underscores the potential for intermetastatic heterogeneity (spatial and temporal) and the need to interpret biomarker results in relation to assay limitations and specimen characteristics (pre-analytical and analytical variability between specimens). Then, the primary tumour had been diagnosed and treated at another institution in 2008; therefore, archival primary tumour tissue was not available at our institution for repeat HER-2 or molecular concordance testing, which represents a limitation of this report.

Brain metastases are believed to spread predominantly haematogenously, although the underlying mechanisms remain incompletely understood (26). Circulating tumour cells have been documented in several malignancies as markers of recurrence risk and poor prognosis, but evidence specifically in GC brain metastases remains limited (26).

Clinically, brain metastases may present with symptoms that markedly impair quality of life, including weakness, headache, gait or balance disturbance, nausea/emesis, focal neurological deficits, seizures, cognitive changes, and ataxia (26, 27). In this case, the solitary brain metastasis presented with a seizure and radiologically mimicked a primary brain tumour, highlighting the diagnostic challenge. Incidental brain metastases are uncommon but may occur, particularly when neuroimaging is performed for surveillance or unrelated symptoms (26, 28).

Management requires integration of neurological symptom control and treatment directed at the primary tumour. Because no specific guidelines for GC brain metastases exist, multidisciplinary collaboration between neuro-oncology, neurosurgery, radiation oncology, pathology, radiology, and gastrointestinal oncology is essential.

For symptomatic brain metastases, treatment options include corticosteroids, surgery, stereotactic radiotherapy, whole-brain radiotherapy, and systemic therapy selected according to the primary tumour profile (29). Surgery followed by postoperative radiotherapy is generally considered for selected patients with good PS, controlled extracranial disease, and solitary or surgically accessible lesions, whereas stereotactic radiotherapy may be preferred when surgery is not feasible and whole-brain radiotherapy is usually reserved for multiple lesions or poor-prognosis settings (30).

In our case, decisions were discussed within both the brain and GC MDTs. After complete resection of the brain lesion and negative systemic restaging, active surveillance was chosen because of the exceptionally long disease-free interval, the absence of extracranial disease, and the lack of evidence supporting pseudo-adjuvant systemic therapy in this setting.

At local intracranial recurrence, stereotactic radiotherapy was delivered for local control. When systemic progression subsequently occurred, modified FOLFOX-4 was selected according to the patient’s age, comorbidities, ECOG PS, nutritional status, stage IV disease, and biomarker profile (HER-2 negative, PD-L1 CPS <1, and MSS), in line with international guidelines available at that time (12).

Systemic treatment for brain metastases remains challenging because drug delivery may be limited by the blood-brain barrier and by efflux transporters such as P-glycoprotein (30). Nevertheless, permeability may vary across metastases, and selected cytotoxic or targeted agents may have activity in specific contexts (30–32). Given the absence of high-level evidence in GC brain metastases, local therapy remains central for symptomatic solitary lesions, while systemic therapy should be individualised according to extracranial disease status and biomarkers.

Overall, the present case should be read as an educational example of diagnostic uncertainty in a very late GEJA relapse rather than as evidence of a previously undescribed metastatic pattern. It highlights three points: very late recurrence remains possible even after a decade without disease; a solitary intracranial lesion can mimic a primary brain tumour and may require tissue confirmation; and interpretation should integrate the biological continuum of GEJA with evidence from both gastric and oesophageal cohorts. The main limitations are the single-case nature of the report, lack of archival primary-tumour tissue for repeat biomarker and genomic concordance testing, absence of paired comprehensive genomic profiling across all tumour sites, and limited generalisability from rare and heterogeneous literature.

## 5 Conclusions

Brain metastases after curative treatment of gastroesophageal adenocarcinoma are uncommon but documented. This report describes a particularly late brain-only presentation thirteen years after curative-intent treatment for a Siewert type II GEJA. Considering the existing literature (33–35), the value of this clinical case is the diagnostic lesson created by the long disease-free interval, the radiological suspicion of a primary brain tumour, negative extracranial staging, and the need for pathological confirmation.

For clinicians, the key message is to consider late metastatic recurrence when a long-term survivor develops new neurological symptoms or a new intracranial lesion, regardless of the duration of prior disease control. However, this observation alone does not provide evidence for routine brain imaging or prolonged intensive surveillance in asymptomatic survivors. Multidisciplinary assessment and tissue diagnosis should be considered when they can meaningfully resolve diagnostic uncertainty and guide treatment. Further research is needed to define the biology, risk factors, and optimal management of brain metastases across the heterogeneous spectrum of GC and GEJA.

## Data Availability

The raw data supporting the conclusions of this article will be made available by the authors on specific request, without undue reservation.
